# The Casualties of War: An Excess Mortality Estimate of Lives Lost in the 2020 Nagorno-Karabakh Conflict

**DOI:** 10.1007/s11113-023-09790-2

**Published:** 2023-05-10

**Authors:** Ariel Karlinsky, Orsola Torrisi

**Affiliations:** 1 The Bogen Family Department of Economics, The Hebrew University of Jerusalem (HUJI), Mt. Scopus, 9190501 Jerusalem, Israel; 2 Division of Social Science, New York University Abu Dhabi, Saadiyat Island, Abu Dhabi, UAE; 3 Department of Social Policy, The London School of Economics, Houghton Street, London, WC2A 2AE UK

**Keywords:** Armed conflict, Excess mortality, Nagorno-Karabakh

## Abstract

*Who and how many died in the 2020 Karabakh War?* With limited evidence provided by authorities, media outlets, and human rights organizations, still little is known about the death toll caused by the 44-day conflict in and around Nagorno-Karabakh. This paper provides a first assessment of the human cost of the war. Using age–sex vital registration data from Armenia, Azerbaijan, and the de facto Republic of Artsakh/Nagorno-Karabakh, we difference the 2020 observed mortality values from expected deaths based on trends in mortality between 2015 and 2019 to offer sensible estimates of excess mortality resulting from the conflict. We compare and contrast our findings with neighboring peaceful countries with similar mortality patterns and socio-cultural background and discuss them against the backdrop of the concurrent first wave of Covid-19. We estimate that the war led to almost 6,500 excess deaths among people aged 15–49. Nearly 2,800 excess losses occurred in Armenia, 3,400 in Azerbaijan, and 310 in de facto Artsakh. Deaths were highly concentrated among late adolescent and young adult males, suggesting that most excess mortality was directly related to combat. Beyond the human tragedy, for small countries like Armenia and Azerbaijan, such loss of young men represents a considerable long-term cost for future demographic, economic, and social development.

**Supplementary Information:**

The online version contains supplementary material available at 10.1007/s11113-023-09790-2.

## Introduction

The territories of Nagorno-Karabakh—a landlocked region that according to international law belongs to Azerbaijan (UN Security Council, 1993a-d), but which Armenia claims to be an integral part of historical Armenian land (Armenia Ministry of Foreign Affairs, 2020)—have been contested by the two countries since the twilight years of the Soviet Union. Since the First Karabakh War (1991–1994) and the creation of the de facto Republic of Nagorno-Karabakh/Artsakh,^1^ Armenia and Azerbaijan have been fighting over the area both in diplomatic venues and militarily on the ground in waves of varying intensity.

On September 27, 2020, after a series of mutual skirmishes, the long-standing dispute turned into a full-scale war, which formally ended only 44-days later with a Russian-brokered ceasefire deal. Known as the Second Karabakh War, the conflict emerged as the worst episode of violence between ethnic Armenian and Azerbaijani forces since the 1994 armistice. It caused severe disruptions to an already isolated and impoverished region and, reportedly, high levels of mortality. During the conflict, both warring parties claimed to have inflicted heavy military losses to their opponent (Heil, 2020; Mitra, 2020). Yet, as the conflict ended, information about casualties among combatants remained sparse and was mostly provided by partisan media outlets and Ministries of Defense, erratically and with a varying degree of transparency (JamNews, 2020). Similarly, information about civilian deaths has been scant to minimal (Amnesty International, 2021).

This paper aims to provide a first account of the extent of excess mortality resulting from the Second Karabakh War, thereby contributing to the long-standing efforts of demographers, statisticians, and medical experts to document the consequences of warfare on population health and mortality (Checchi et al., 2022; Murray et al., 2002; Tabeau & Bijak, 2005). The disarray and chaotic environment that conflict creates generally leads to the disruption of systems of data collection. Hence, most prior literature has relied on retrospective surveys or pre-post census data to estimate changes in mortality due to war and armed violence (Burnham et al., 2006; de Walque, 2005; Hagopian et al., 2013; Heuveline, 1998; Levy & Sidel, 2016; Spiegel & Salama, 2000). In most cases those data sources provide the best-of-the-worst option, but they also often suffer from sampling, recall and selection bias, surveys in particular (Checchi et al., 2022; Working Group for Mortality Estimation in Emergencies, 2007). As information are typically collected long after the end of hostilities, researchers can rarely provide prompt evidence on conflict-caused mortality (Checchi & Roberts, 2008), and in some instances different semantics and analytical methodologies have led to polarizing debates (Hagopian et al., 2013, 2018; Spagat & van Weezel, 2017). Although no comprehensive assessment of the severity of a conflict is possible without knowledge of its impact on human life, estimating war deaths is notoriously difficult, sensitive and can be remarkably contentious (Guha-Sapir & D’Aoust, 2011).

In this study, we take advantage of civil registration data in Armenia, Azerbaijan, and de facto Artsakh, which have improved considerably in terms of coverage and registration in the twenty-first century (UN ESCAP, 2020a-b; Mikkelsen et al., 2015; Wang et al., 2017; WHO, 2006) and were made more readily and openly available during the pandemic than in earlier periods. Importantly, vital registration systems remained fully operational during the 2020 Covid-related lockdowns and the conflict in all three territories (AbouZahr et al., 2021). We thus employ age–sex-disaggregated data on death counts and compute the 2020 expected number of deaths on the basis of mortality over the period of 2015–2019 in each population. We then estimate excess mortality as the difference between the observed 2020 mortality and the expected value for men and women separately and for diverse age groups.

We estimate that among people aged 15–49, the conflict overall led to nearly 6,500 excess deaths, i.e., deaths that would have not occurred in the absence of conflict violence. Specifically, we estimate about 3,400 excess deaths in Azerbaijan, 2,800 in Armenia, and 310 in de facto Artsakh. Age–sex-disaggregated analyses show that in all three territories excess mortality was largely concentrated in groups with low risk of first wave of Covid-19 mortality (Goldstein & Lee, 2020; O’Driscoll et al., 2021), but very likely to be actively engaged in fighting. Out of the total excess mortality burden, more than a quarter was among young adult men (aged 20–24) in Azerbaijan and de facto Artsakh. In Armenia, deaths among male adolescents (15–19) constituted nearly 38% of the estimated excess mortality. Here, where data allow us to examine death counts by week, we also show that the observed peaks in male mortality coincided with the conflict months (September–November 2020). We observe no similar changes for same-age (or older) women. No comparable increases occurred in other causes of death typically affecting young and middle-aged adults (e.g., road traffic accidents or homicides), and in populations with similar pre-2020 mortality levels, socio-economic and cultural background, such as neighboring and more peaceful Georgia, Iran, and Russia.^2^ This evidence together helps us to attribute our numbers more confidently to the war and fighting activities than to other causes, including Covid-19.

Our findings suggest that in all three belligerent populations, excess mortality was highly selective and mostly due to direct combat. While this implies that the mortality toll on civilians was relatively modest compared to other current conflicts, such as Ethiopia, Syria, Yemen, or Ukraine, for small countries like Armenia (3 million) and Azerbaijan (10 million), this high number of “deaths in uniform” represents remarkably heavy losses, especially given the short duration of the war. Besides the human tragedy, the premature loss of many men in young cohorts represents a potentially large long-term cost for the socio-economic development of both Armenia and Azerbaijan and a threat to social stability in an already fragile region.

## Short History of the Nagorno-Karabakh Conflict

The conflict between Armenia and Azerbaijan over Nagorno-Karabakh is the longest-running unresolved dispute in the ex-Soviet space, tracing its roots to the last years of the USSR and its structural arrangements. During the Soviet era, the region was granted an autonomous status—the Nagorno-Karabakh Autonomous Oblast (NKAO)—within the then Soviet Socialist Republic (SSR) of Azerbaijan, but its borders contained a sizable Armenian population (de Wall, 2003; Demoscope Weekly & USSR Population Statistical Collection, 2016). In 1988, as Gorbachev’s *glasnost* began to allow popular expressions of grievances, NKAO asked Soviet authorities in Moscow to be transferred from the Azerbaijani to the Armenian SSR. The request ignited violent unrest between the two SSRs, which escalated into an inter-state conflict as Armenia and Azerbaijan gained their independence. In December 1991, Armenia-backed separatists from Nagorno-Karabakh seceded from Azerbaijan, marking the start of the First Karabakh War (Cornell, 2011, 2017; de Waal, 2003).

This first confrontation officially ended with a ceasefire in 1994 and resulted in the creation of a self-proclaimed entity—the Republic of Nagorno-Karabakh or Artsakh. The Republic has since operated as an independent state with its own legislature, executive, and judiciary authorities and has received support and protection from Armenian military forces. However, it has never been recognized by any foreign government, including Armenia. Together with territories of de facto Artsakh,^3^ ethnic Armenians gained control and populated the Kelbajar-Lachin region,^4^ the Jabrail district, and the Western parts of Agdam, Fizuli and Terter. Altogether these territories comprise approximately 20% of Azerbaijan’s internationally recognized territory (UN Security Council, 1993a-d). While no consolidated figure has been produced on the casualties of the First Karabakh War, historical sources and international organizations estimated that between 17 and 30,000 people lost their lives (Cornell, 2017; de Wall, 2003; Human Rights Watch, 1994; Yunusov, 2002). Nearly 700,000 Azerbaijani from Nagorno-Karabakh or Armenia and more than 350,000 Armenians from Azerbaijan were displaced (Human Rights Watch, 1994; UNHCR, 2009a-b).

Since the 1994 ceasefire, Armenia and Azerbaijan have continued the fight over the territory in waves of varying intensity. Incidents along the line of contact resulting in regular deaths have been reported each year (for examples see International Crisis Group (2022)). Of note, particularly violent hostilities happened over four days in April 2016, when reportedly 200 people died (U.S. Department of State, 2016).

**Fig. 1:**
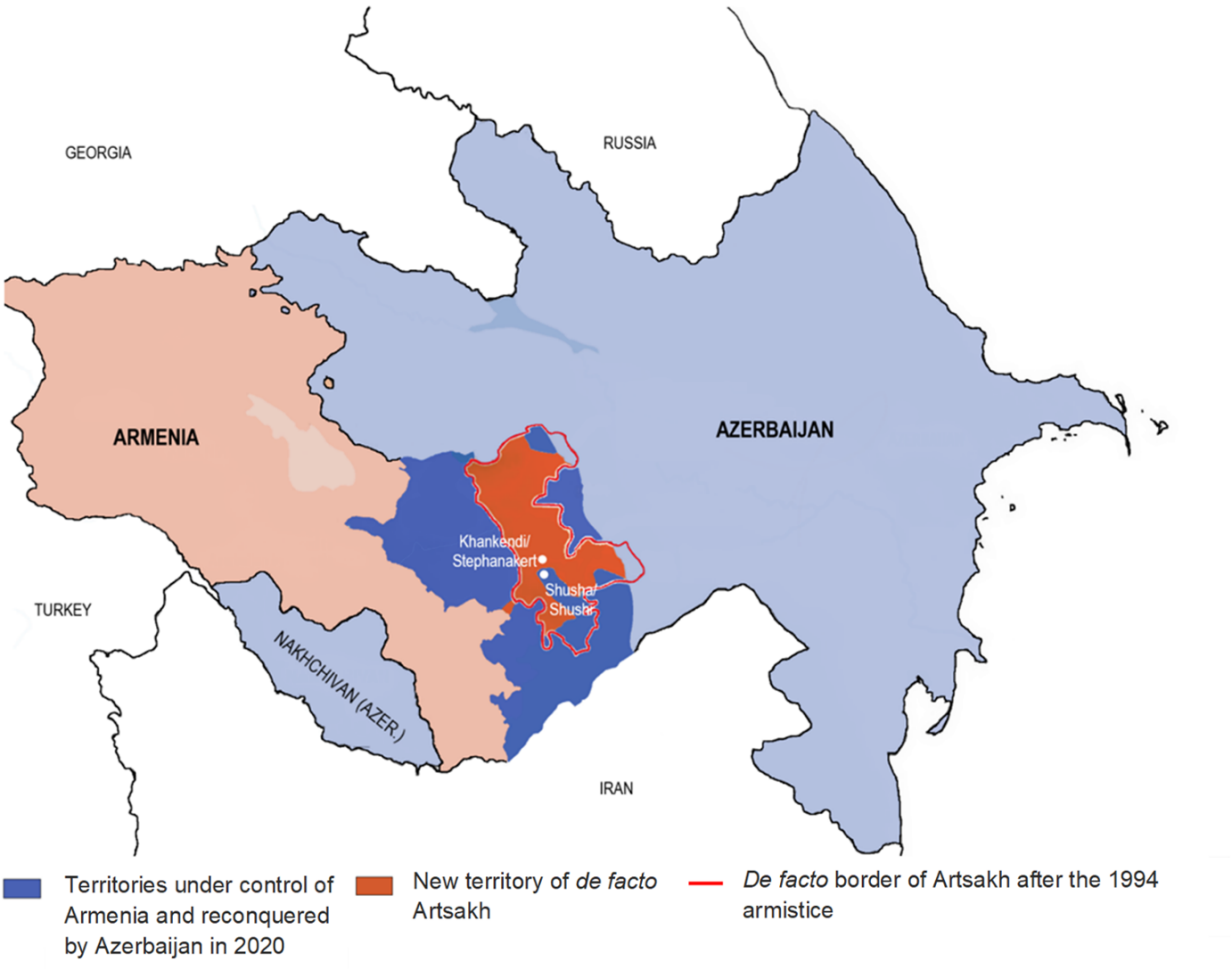
Post-2020 ceasefire map of Nagorno-Karabakh. Source: DIVA-GIS (2022) for shapefile

To date, the most intense confrontation since the 1994 ceasefire began in the autumn of 2020. In late September, while the Covid-19 pandemic distracted the international community and constrained diplomacy, serious fighting between Azerbaijani forces and Armenian-backed Karabakhis broke out. In mid-October, Azerbaijan gained control of the strategic town of Hadrut inside Artsakh and later reconquered the Lachin Corridor, the connecting route between Armenia and Artsakh, and the town of Shusha/Shushi (de Waal, 2021). On November 9, 44 days after the start of the war, Armenia and Azerbaijan signed a Russian-brokered ceasefire, which determined considerable military setbacks for Armenians and a significant shift of territorial control in favor of Azerbaijan (Fig. 1) (de Waal, 2021).

Major losses were reportedly suffered by both warring factions. During the conflict, respective authorities made a number of public claims about military losses inflicted to the opponent.^5^,^6^ By contrast, information about incurred losses were vague, especially in Azerbaijan where the martial law imposed by the government largely restricted access to information (Heil, 2020). International organizations attempted with difficulty to document atrocities on both sides (Amnesty International, 2021; Human Rights Watch, 2020a). Only months after the ceasefire, the belligerents released some information about military casualties. Yet, again, these mostly came as scattered snippets on partisan media outlets and their credibility has been contested (JamNews, 2020). Media reports of data obtained from the Armenian Investigative Committee stated that 3,822 Armenians, including soldiers and civilians, died in the conflict and 208 were still missing as of March 2022 (ARKA News, 2022; ArmenPress, 2022). The Azerbaijani government did not release any official data on civilian casualties, but in July 2021 published a list of 2,906 confirmed dead and 8 missing soldiers (Azerbaijan Ministry of Defense, 2021). The Human Rights Ombudsman of Artsakh reported to the UN General Assembly that 80 Karabakhi civilians—68 males and 12 females—were killed in the territories of de facto Artsakh (UN General Assembly, 2021). Aside from these data and from two reports by non-governmental organizations (Amnesty International, 2021; International Crisis Group, 2022), which claimed that around 170 civilians died as a result of indiscriminate strikes, no other independent effort has been made to document the mortality burden caused by the war.

## Data and Methods

Our main source of data is official reporting on death counts registered from 2015 to 2020 by National Statistical Committees in Armenia, Azerbaijan, and de facto Artsakh. In Armenia, we use information from the National Demographic Yearbook (Statistical Committee of Armenia, 2021) and from Eurostat’s special collection of weekly total death counts by age and sex, which began in May 2020 “to support the policy and research efforts related to Covid-19” (Eurostat, 2021). In Azerbaijan, the main source of data is the Demographic Yearbook and the online spreadsheet on total deaths by age and sex (Statistical Committee of Azerbaijan, 2021a-b). The data is annual, with monthly data only available for total deaths, i.e., without age or sex desegregation (Karlinsky & Kobak, 2021). For de facto Artsakh, we employ data from the National Statistical Service of Nagorno-Karabakh (2021). These are historically collected separately from Armenian statistics (Duthé et al., 2010; Rowland, 2008). For further information on data availability and quality, see the Supplementary Material.

Our method contrasts deaths during 2020 with the expected number of deaths, based on historical number of deaths. The “expected deaths” represent the counterfactual number of deaths that would have occurred under normal circumstances, i.e., in the absence of high mortality events such as conflict (or a pandemic). We derive the expected number of deaths for each population, disaggregated by age groups and sex. Specifically, we utilize age–sex death counts from 2015 to 2019 across 16 age groups^7^ for both males and females. Note that in our main results, we focus our visualizations on individuals aged 0–59 and mostly discuss excess mortality between age 15 and 49 as a first basic strategy to ‘purge’ the estimates as much as possible from the confounding effect of the Covid-19 pandemic, which is known to have had its strongest mortality consequence on individuals aged 60 + (O’Driscoll et al., 2021). In the Supplementary Material, we provide model predictions for all ages (Tables A1-3). Formally, let *c* denote country, *s* denote sex, *a* denote age group, and *t* denote year, we estimate:

1 $$Excess\,Deaths_{c,s,a,2020} = Observed\,Deaths_{c,s,a,2020} - Expected\,Deaths_{c,s,a,2020}$$

where

2 $$Expected\,Deaths_{c,s,a,2020} = f\left( {Observed\,Deaths_{c,s,a,2015 - 2019} } \right)$$

We estimate three variations of $f\left( {Observed\,Deaths_{c,s,a,2015 - 2019} } \right)$, using the following:

1. Over-dispersed Poisson mean (OPM)
2. Over-dispersed Poisson trend (OPT)
3. Lee-Carter model

The first two models assume that deaths follow an over-dispersed Poisson distribution.^8^ The OPM estimate is the mean number of deaths from 2015 to 2019, while the OPT estimates a linear trend across 2015–2019, as the mean number of deaths might miss an increase or decrease in deaths due to various reasons such as changes in underlying health, external conditions (e.g., better/worse traffic safety), age composition within age groups, etc. Finally, the Lee-Carter (1992) model, which is adapted to mortality counts (rather than rates) and was kindly provided to us by Schöley (2022), forecasts the 2020 expected deaths for each population by considering the entire matrix of age–sex–year death counts instead of forecasting separately for each age–sex group. In addition, since we may be concerned about potential migration, which could reduce exposure to violence and deaths during war, and given that prior research has shown that the estimation of excess mortality may be sensitive to modeling decisions (Nepomuceno et al., 2022; Schöley, 2021), as a check, we estimate the three models using death rates rather than counts for the two main belligerents—Armenia and Azerbaijan.

We combine the outputs of the three models to arrive at our final prediction point estimate and confidence interval. We take the highest point-estimate across the three models, as well as the lowest lower bound and the highest upper bound as our final estimate for each country–age–sex.^9^ These represent the most conservative estimate and surrounding confidence interval of these three models (also known as an “enveloping interval,” see Gaba et al. (2017)), thus minimizing false-positives (i.e., finding excess where there is none). In practice, the differences between the predictions from these models are small such that other combinations will have similar results.^10^ As shown in Eq. (2), excess deaths are then derived as the difference between the observed number of deaths in 2020 and our final expected point estimate, with the excess confidence interval derived by subtracting the observed from the expected confidence interval bounds.

## Results

We begin by showing our results for the territories directly involved in the Second Nagorno-Karabakh war (Armenia, Azerbaijan, and de facto Artsakh). Next, we discuss potential alternative explanations and compare the results with neighboring countries that did not experience conflict in 2020.

### Armenia

**Fig. 2:**
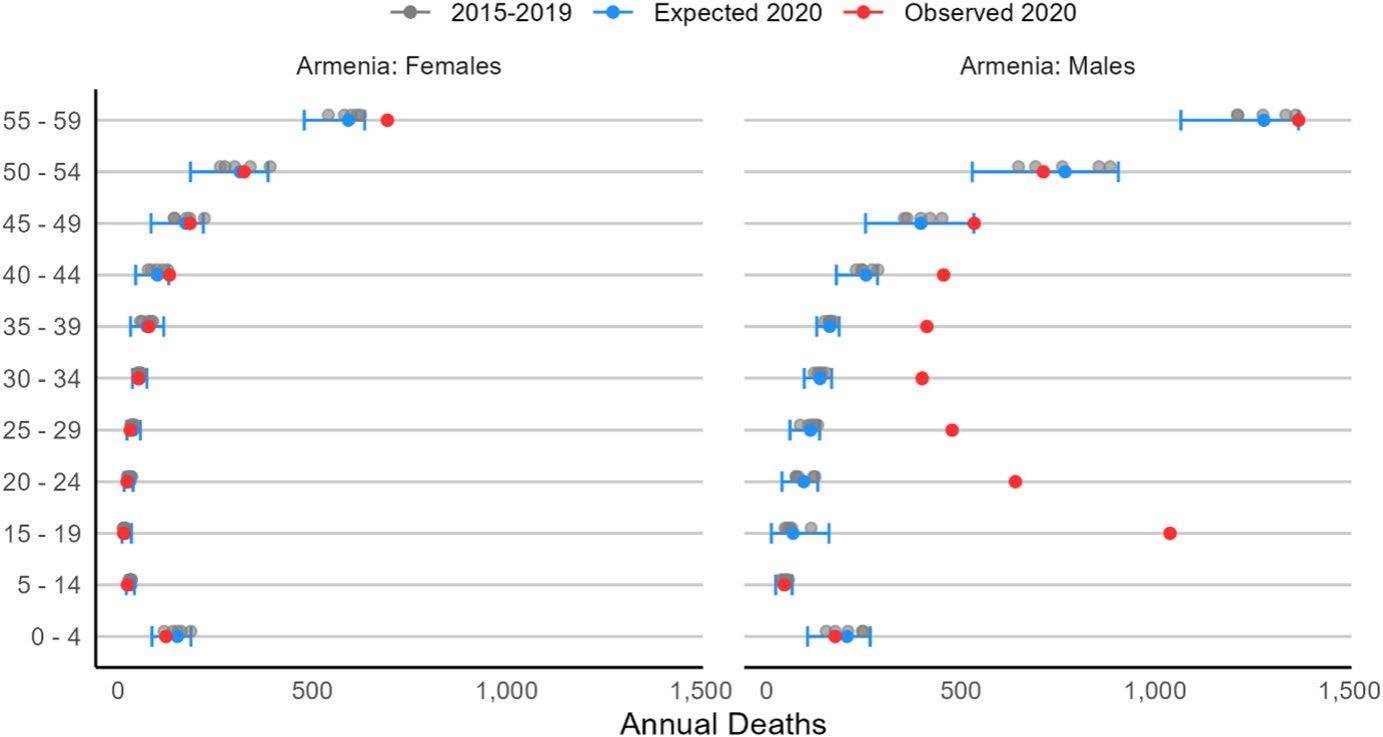
2020 Expected and observed mortality by age group and sex, Armenia. Source: Armenia National Demographic Yearbook (Statistical Committee of Armenia, 2021)

In Fig. 2, we show, in blue, the model-estimated expected number of deaths for 2020 in Armenia by age group and sex and, in red, the observed number of deaths. In gray, we also report the raw death counts for the years 2015–2019, i.e., the data points used to predict the expected 2020 mortality values for each age–sex combination.

For females, observed deaths are well within the bounds of expectation across most age groups, with excess mortality visible only for women aged 55 + . That is, Armenian female mortality in 2020 does not appear to be significantly affected by the conflict (or other causes), and some excess mortality is observed only in late mid-life. These excess losses among older-aged women are likely attributable to the first wave of Covid-19, which hit hardest on individuals nearing their 60 s and above (O’Driscoll et al., 2021). We return to this point later.

No considerable excess mortality is observed for male children and young boys (ages 0–14). By contrast, there is a stark increase in deaths among Armenian men aged 15–44. For these age groups, our observed values are all well above the upper bound of the confidence intervals. The most dramatic increase from expected deaths—both in absolute and in relative terms—is observed for the 15–19 age group, which suffered 1,038 observed deaths compared to an expectation of less than 70 [Enveloping Interval: 11, 160]. This represents nearly 38% of all excess mortality in Armenia among those aged 15–49. At older ages, the trend of higher observed mortality than expected continues, but is generally decreasing with age: between ages 45 and 59, observed deaths align back within bounds of expectation and we observe excess mortality again only in the oldest age groups (60 +), as for females (see Tables A1). This is again likely the result of excess mortality due to the pandemic, not the war.

**Table 1:** Total excess mortality by country and sex, age groups 15–19 to 40–49

	Female	Male	Total
Armenia	27 [− 153, 270]	2730 [2358, 3187]	2757 [2205, 3457]
De facto Artsakh	− 1 [− 60, 21]	314 [251, 363]	313 [191, 384]
Azerbaijan	260 [− 233, 783]	3121 [2319, 4071]	3381 [2086, 4854]
Total	286 [− 446, 1073]	6165 [4928, 7621]	6451 [4482, 8694]

Note: Enveloping excess bounds are in square brackets. Sources: Armenia National Demographic Yearbook (Statistical Committee of Armenia, 2021); Azerbaijan Demographic Yearbook (Statistical Committee of Azerbaijan, 2021a-b); National Statistical Service of Nagorno-Karabakh (2021)

**Fig. 3:**
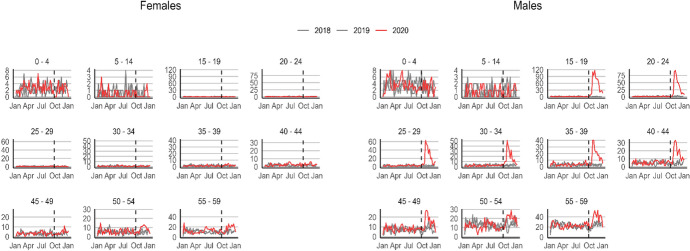
Weekly deaths by age group and sex, Armenia. Source: Eurostat (2021). Note: the dashed line indicates September 27, 2020 (war onset). The y-axes differ across age groups, but are the same for men and women belonging to the same age category

To better evaluate this claim and provide additional evidence for Armenia, we utilize weekly registration data on deaths by age and sex from Eurostat (2021). This database was originally set up in May 2020 to assist research efforts on Covid-19 mortality and, among the belligerent countries, is available for Armenia only. It is worth noticing that here data for Armenia is provided by date of death registration. In Fig. 3, we plot weekly registered deaths by age groups and sex as reported until December 2020. Since not all conflict deaths were registered during the conflict, with some being registered only in 2021 up to the end of March (Statistical Committee of Armenia, 2021), in Figure A2 in the Supplementary Material, we further add deaths registered between January and March 2021 for completeness and transparency. In either Figure, for men aged 15–49, the uptakes in mortality coincided fairly precisely with the onset and development of the conflict (September–November 2020). No similar increase or deviation from the preceding year is visible for women, male children, and young adolescents. Also, no similar pattern emerges in Georgia, a more peaceful country in the region for which Eurostat data are available and comparable (see Figure A3 in the Supplementary Material).

### Azerbaijan

**Fig. 4:**
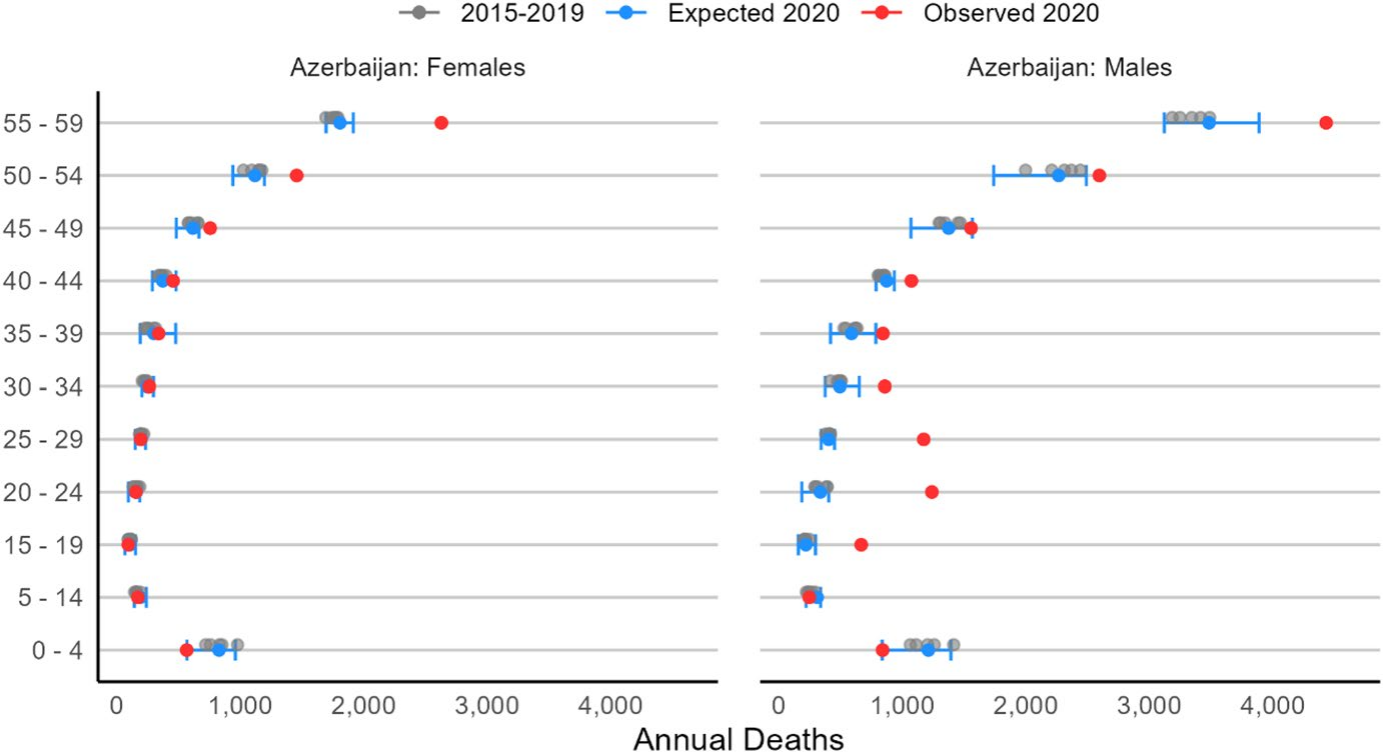
2020 Expected and observed mortality by age group and sex, Azerbaijan. Source: Azerbaijan Demographic Yearbook (Statistical Committee of Azerbaijan, 2021a-b). Note: the x-axis ranges from 0 to 5,000

In Fig. 4, we show the results for Azerbaijan. The pattern is similar to Armenia: observed deaths fall largely within the bounds of expectation at very young ages (0–14) for both men and women, and female excess mortality is observed only after age 49. Again, this latter result is likely due to the Covid-19 pandemic, whose first-wave impact was heavily concentrated among older adults.

By contrast, we observe a significant increase in observed mortality—well-above expectation and its upper-bound confidence interval—for males between the ages of 15 and 44, and even up to 49, where the observed mortality value is much higher than the expected, but falls within the predicted confidence interval. Slightly different from Armenia, in Azerbaijan, the bulk of excess mortality is among young adult males (aged 20–24), with 1,240 observed deaths compared to the 337 [188, 405] we would have expected to observe in 2020. This is broadly consistent with data provided in the July 2021 list of deceased male soldiers released by Azerbaijan’s Ministry of Defense (2021), in which the median age at death of the reported victims is 24 years old (*mean:* 25.4, *s.d.* 5.9).

### Artsakh

**Fig. 5:**
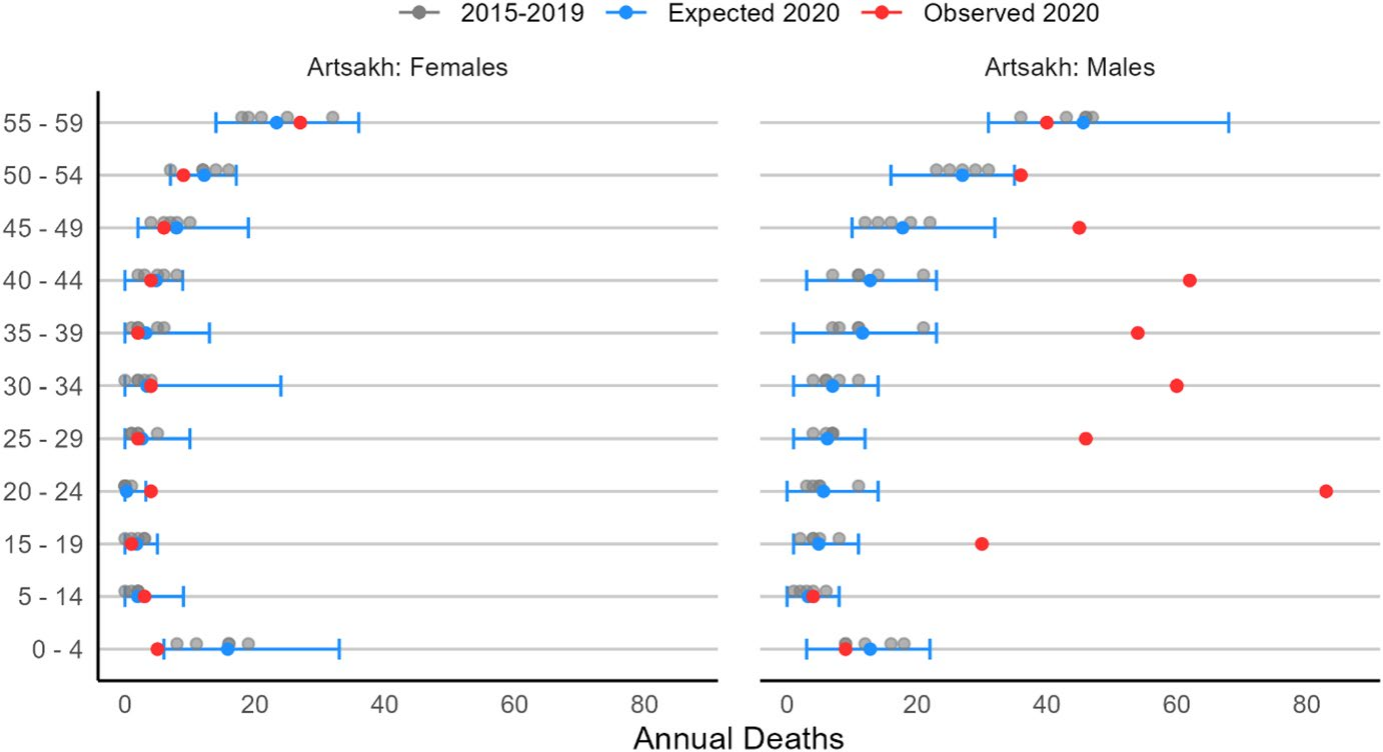
2020 Expected and observed mortality by age group and sex, Artsakh. Source: National Statistical Service of Nagorno-Karabakh (2021). Note: the x-axis ranges from 0 to 90

Figure 5 shows the results for de facto Artsakh which, while supported by Armenia, operates its own independent vital registration system and has its own military force (Duthé et al., 2017; Cornell, 2017; de Wall, 2003). Owing to its small population size, the group-wise total and expected deaths in de facto Artsakh are noisier and have relatively wide confidence intervals. Nevertheless, the pattern resembles closely those of Armenia and Azerbaijan.

We find no evidence of excess mortality among women at all ages, as well as among boys (0–14). Conversely, a shift from expected to observed deaths is again visible for men in their late teens and up to age 49. As in Azerbaijan, the most significant increase in mortality is among men of combat age (20–24), with 83 observed deaths compared to the 6 [0, 14] we would have expected in 2020.

Overall, in all belligerent populations, individuals aged between 15 and 49 suffered the largest excess mortality in 2020. Thus, to provide a comprehensive figure of excess deaths in the Second Karabakh War, we sum the estimated excess mortality in each population-sex across these age groups. The results are presented in Table 1. As the Figures above already shown, the vast majority of excess deaths occurred among men. For them, both the point estimates as well as the excess bounds are all positive in each country and in total sum. For females, while we find some excess losses in Armenia and Azerbaijan, these are statistically non-significant, both at the country level and in total. Overall, our best estimate is that excess mortality for individuals aged 15–49 amounted to 6,451 [4482, 8694] extra deaths, with over 95% occurring to men. Armenian deaths constituted about 43% of the toll, Azerbaijani nearly 52% and the remaining 5% were observed in de facto Artsakh.

It is important to note here these estimates represent the total excess mortality in 2020 in the three populations and include both direct violent deaths (e.g., deaths occurred during active combat) and conflict-related indirect mortality (e.g., due to infrastructure or healthcare service collapse/unavailability). We are unable to difference out this total excess between direct and indirect mortality. Yet, we posit that most of the excess losses we estimate are related to direct deaths among fighters and that indirect effects are small. This is because, had indirect mortality been more pronounced, we would have expected to observe a larger, or at least some, excess mortality among women and children as typically found in other war scenarios (Hagopian et al., 2013; Tabeau & Bijak, 2005).

As highlighted earlier, there are two other possible concerns with our estimates. First, another episode of violence occurred in 2016. Although this was very short in time (about 4 days), it is important to check if including 2016 affects our expected mortality estimate for 2020. Hence, we further re-estimated the models using only with information from the years 2015, 2017–19. Reassuringly and expectedly, since violence in 2016 was much more limited than in 2020, results remain practically unchanged.^11^

Second, there is the possibility that not all the estimated excess mortality is solely attributable to the conflict, but may also reflect increased mortality from other causes, particularly the Covid-19 pandemic, which caused massive excess mortality across the world, including the Caucasus region (Karlinsky & Kobak, 2021) as well as from prevalent causes of death among young and middle-aged adults (e.g., road traffic accidents, homicides). Unfortunately, neither Armenia’s nor Azerbaijan’s Demographic Yearbooks provided sufficient age–sex-disaggregated data to decompose trends by causes of death for 2020, as well as for the preceding five-year window. Moreover, while Armenia categorized separately deaths due to war violence (in aggregate format only), Azerbaijan did not differentiate them from other ‘external causes’ (that is, it counts them together with road accidents, poisoning and injuries). The cause-of-death attribution might also be unreliable for political reasons, especially in authoritarian and conflict-affected regimes (García & Aburto, 2019; Khorram-Manesh et al., 2021). These different coding procedures, issues, and data availability make it hard to systematically analyze and compare changes across and within populations. Despite the lack of finer-grained data, we nevertheless examine changes in the typical leading causes of death in older adolescents and young adult men—road traffic accidents and homicides—which could provide an alternative explanation to our estimated excess mortality values, using available aggregate-level data (i.e., not disaggregated by age groups) for the 2015–2020 period provided by the United Nations Economic Commission for Europe (UNECE) (2022) for road traffic accidents and by the United Nations Office on Drugs and Crime (UNODC) (2022) for homicides. We find no significant changes in 2020 compared to previous years in either cause of death, indicating that the excess mortality we observe is not due to these factors (see Figure A1 in the Supplementary Material). In addition, our general results did not change when we estimated the models on death rates.^12^

With regards to Covid-19 pandemic, again we are unable to neatly identify the extent to which the estimated excess mortality is due to Covid-19. However, the facts that (i) most excess mortality occurred at ages that were at low risk of Covid-19 mortality, at least in the first 2020 wave (O’Driscoll et al., 2021) and that (ii) at least in Armenia, we have evidence that the increase in mortality, especially for men aged 15–49, coincided with the development of the conflict, provide good grounds to believe that most of our estimated excess mortality is related to the war. As an additional way to explore this aspect and attribute our numbers more confidently to the war and fighting activities than to the pandemic, we next examine whether any comparable increase occurred in the countries and populations neighboring the belligerents, but which did not experience a conflict in 2020.

### Comparison to Neighboring Countries

**Fig. 6:**
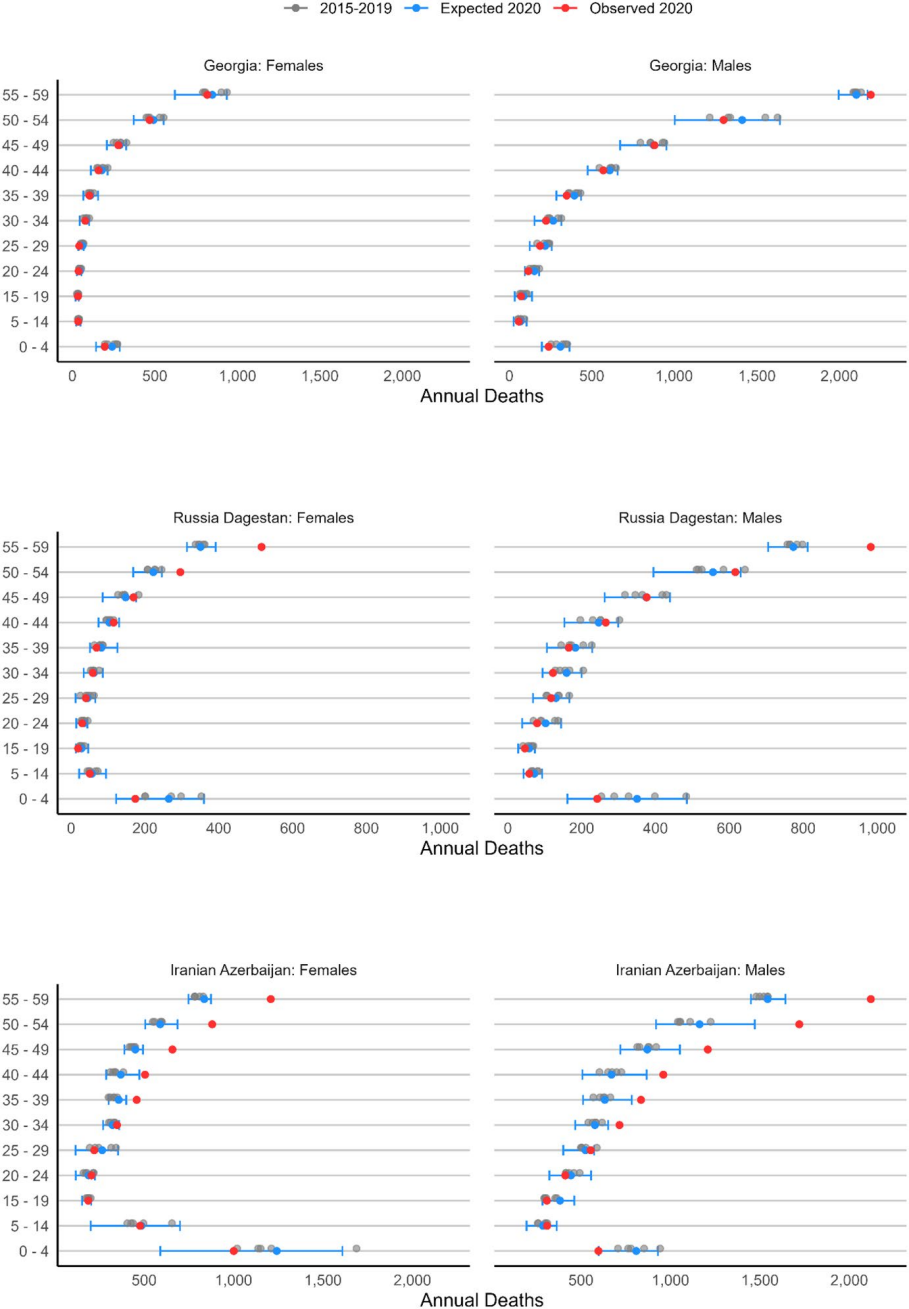
2020 Expected and observed mortality by age group and sex, neighboring countries/territories. Sources: GeoStat (2021) for Georgia; Ghafari et al. (2022) for Iran. For Russian, Dagestan data were kindly supplied by Dmitry Kobak

Armenia and Azerbaijan are neighbored (clockwise) by Georgia, Russia’s Dagestan region, Iran’s Azerbaijan region (comprising three provinces with significant Azeri population) and Turkey (Fig. 1). None of these population experienced armed violence in 2020. We thus estimated the same models as for the belligerents, using age–sex vital registration data from Georgia (GeoStat, 2021), Russia^13^, and provinces of Iran (Ghafari et al., 2022).^14^ The results of our analyses are in Fig. 6.

Estimates from neighboring countries are consistent with the hypothesis that the vast majority of young male deaths in Armenia, Azerbaijan and de facto Artsakh are mostly due to the conflict rather than to other causes such as Covid-19. In both Georgia and Dagestan there is virtually no excess mortality in 2020 except for men aged 55–59 (and again, expectedly for individuals aged 60 +), let alone from 15–19 to 40–49. In Iran’s Azerbaijan region, we do not observe any excess mortality for adolescent and young adults, either male or female. Only around ages 30–34, there is some, yet small excess mortality, and it is not comparable in magnitude to that observed in the three warring populations. Iran has been one of the earliest and hardest hit countries by the pandemic both regionally and globally (Ghafari et al., 2022). Hence, this slightly higher excess mortality at younger adult ages—which, in contrast to Armenia and Azerbaijan, is here visible in both men and women—is likely attributable to the first wave of the pandemic. Overall, summing up the excess in the neighboring countries aged 15–44, both among males (606 [-872, 2759]) and females (377 [-324, 1384]) excess mortality estimates are not statistically significant. They are also non-significant at the country level.

Our estimates of excess mortality in more peaceful countries bordering Armenia and Azerbaijan thus suggest that the Covid-19 pandemic in the region, which is the most significant concurrent excess mortality factor in 2020 to the conflict, led to almost no excess deaths at the ages we observed it for Armenia, Azerbaijan and de facto Artsakh. This indicates that most, if not all the excess mortality we estimated (Table 1) is likely directly attributable to the conflict.

## Discussion and Conclusion

Mortality, both among civilians and soldiers, is undoubtedly the primary indicator for assessing the severity and disruptiveness of warfare on human societies (Checchi & Roberts, 2008). Reliable casualty estimation is also essential to understand the demographic sequelae of armed conflict, to select and adjust relief operations, and is important for documentation purposes (Checchi et al., 2022; Guha-Sapir & D’Aoust, 2011). In this study, we contribute to the demographic and public health literature concerned with conflict casualty estimation (Checchi et al., 2022; Hagopian et al., 2013; Khorram-Manesh et al., 2021, 2022; Tabeau & Bijak, 2005) by providing the first independent assessment of the mortality impact of the 2020 Second Karabakh War.

We estimate that nearly 6,500 excess deaths have occurred in relation to the conflict. In specific, we estimate about 2,800 excess deaths in Armenia, 3,400 in Azerbaijan, and 310 in de facto Artsakh among people aged 15–49. Our age–sex-disaggregated analyses indicate that excess mortality involved preeminently adolescent and young adult men, a demographic group which was not severely hit by the concurrent first wave of the pandemic (Goldstein & Lee, 2020; O’Driscoll et al., 2021), but highly likely to be actively engaged in fighting. We did not find any increase in ‘external’ causes of death other than conflict which could alternatively explain the upticks in mortality for this group, nor similar patterns of excess losses in neighboring and more peaceful countries. We also showed that in Armenia the rise in weekly death counts coincided fairly precisely with the violent months of September–November 2020, and that in Azerbaijan, our most-affected ages agree with the distribution of ages at death in the government’s list of deceased servicemen. We take these finding as suggesting that most excess deaths occurred among soldiers and were combat-related, likely resulting from exchanges of fire, missiles, “kamikaze” drones and, among others, the documented use of unlawful cluster bombs (Amnesty International, 2021).

The seemingly limited mortality effect of the war on civilians is in line with available evidence gathered at different times by non-governmental and human rights organizations (Amnesty International, 2021; Human Rights Watch, 2020b; International Crisis Group, 2022) and should not come as a surprise in this context. First, differently from other current theaters of violence, such as Syria or Ukraine, where conflicts have taken a tremendous death toll on civilian populations (United Nations High Commissioner for Human Rights, 2022a-b), the second Karabakh War was of high intensity, but short duration. Second, surely the conflict contained some elements of modern urban warfare (e.g., the fight for control of the city of Shusha/Shushi), which is known to put civilians at high risk of violent death (Moser & McIlwaine, 2014; UN Security Council, 2022). However, the disputed territories in and around de facto Artsakh are predominantly rural, and were already low in population size and density before the war, following decades of low fertility and high out-migration rates. In fact, according to the latest Census, the enclave counted 150,932 residents in 2015, leading to about 12 inhabitants per km^2^ (National Statistical Service of Nagorno-Karabakh, 2016). Of these, between 14 and 17,000 were estimated to live in the adjacent occupied territories of Azerbaijan (International Crisis Group, 2019; OSCE 2011). These conflict and territorial features likely prevented the conflict from having the dramatic and severe impacts on civilian mortality observed elsewhere. While this is somehow our ‘least negative’ finding, it is well known that armed conflict has myriad other consequences on civilians, beyond immediate mortality. Physical and mental health traumas, displacement, consequences for reproductive health, family choices, and bereavement, to name a few, have been documented in many conflict-affected settings, including in relation to the First Karabakh War (Alburez-Gutierrez, 2022; Kerimova et al., 2003; Mavisakalyan & Minasyan, 2021; Murray et al., 2002; Torrisi, 2020; van Baelen et al., 2005; Williams et al., 2021). Additionally, many civilians as well as combatants remain wounded and suffer long-term disabilities. For soldiers, the most recent estimates suggest that for each death, between 3 and 10 combatants are wounded (Fazal, 2014). For the general population, novel studies indicate that war leads to significant losses in disability-adjusted life years (DALYs) resulting from increases in years of life lost (YLLs) attributable to conflict-related injuries (Jensen et al., 2021). These other outcomes should be given serious consideration in future research on the case and in other current war scenarios such as Ukraine, especially in light of the ‘double burden’ of exposure to deadly violence and the Covid-19 pandemic, and the known interaction between war and infectious diseases (Price-Smith, 2020; The Lancet Infectious Diseases, 2022). In this respect, commentators have already highlighted that the Second Karabakh War may have heightened the risk for Covid-19 infections among civilians and made it harder for those affected, including active soldiers, to abide by health guidelines and protective behaviors (Balalian et al., 2021).

While the mortality impact of the Second Karabakh War on civilians seems limited, we found that excess deaths were highly concentrated among adolescent and young adult males, most likely killed in-action. Although data did not allow to carry out more age-disaggregated or cause-specific analyses, in Armenia, more than a third of excess deaths among those aged 15–49 in 2020 concerned men in their late adolescence. In Azerbaijan, more than a quarter involved young males aged 20–24. Put into perspective, these excess mortality values are almost equivalent to 11 years of expected deaths for late adolescents in Armenia and 3 years for young adult men in Azerbaijan. The loss of this many young men at the front in just six weeks of conflict, combined with historically high male-emigration rates and the Covid-19 pandemic (Dermendzhieva, 2011; Karlinsky & Kobak, 2021; World Bank, 2019; WHO, 2022), could represent a serious concern for social stability and for many other social and economic dimensions, including household welfare, labor, and marriage markets.

In addition, while it is not our role to provide normative judgements on whom nation states recruit and send to fight, these figures should at least cause reflection among military and government circles over the fighting preparedness and competence of those sent to the front.^15^ Besides being a morally contentious issue in the ethics of war (Pattison, 2012), leaving the brunt of the fighting to conscripts and very young volunteers is militarily inefficient and has been suggested as one of the primary reasons behind Armenia’s defeat (Amirkhanyan, 2022; Stronel, 2021).

When studying conflict-related mortality, the primary concern and challenge is data quality, coverage, and availability. In this study, we derived our estimates using official vital registration data from Armenia, Azerbaijan, and de facto Artsakh. Typically regarded as “the gold standard” data source for monitoring mortality, the recording of such data may be already poor in conflict-affected areas, become dysfunctional or cease to operate completely in wartimes (Burkle & Garfield, 2013; Checchi & Roberts, 2008). Over the first decades of the twenty-first century, civil registration systems have improved significantly in the South Caucasus both in terms of registration and coverage, and particularly in Armenia (Mikkelsen et al., 2015; UN ESCAP, 2020a; Wang et al., 2017). Importantly, the registration of vital events continued throughout the Second Karabakh War in all three territorial entities. Perhaps because of the need to monitor trends and patterns of Covid-19, the data were also accessible to researchers, something that should not be taken for granted in conflict settings and in countries with secretive governments like Azerbaijan (García & Aburto, 2019; Khorram-Manesh et al., 2021). We have performed a number of checks and do not expect the data errors more prevalent in Armenian and Azerbaijani statistics—under-registration of deaths at extreme ages (Duthé et al., 2017; Mikkelsen et al., 2015)—to compromise our results (at most, they would lead to an underestimation of excess mortality), or other causes besides conflict to majorly distort our estimates. Nevertheless, even in this ‘positive’ data scenario, we are aware that mortality information can be prone to manipulation and/or inaccuracies. Increasing household survey data collection (e.g., only one Demographic and Health Survey was conducted in Azerbaijan in 2006, and in Armenia the last was in 2015) and exerting more pressure on government agencies to release more micro-level census data in the future would be essential to evaluate mortality trends and patterns in the region, and to further unpack the effects of this tragic, and yet unresolved conflict.

## Supplementary Information

Below is the link to the electronic supplementary material.

Supplementary file1 (DOCX 663 kb)

## Footnotes

1 In the sake of space and to avoid confusion, throughout the paper we refer to the territories of the de facto Republic of Nagorno-Karabakh with the term de facto “Artsakh”.

2 Note that the analyses precede Russia’s invasion of Ukraine.

3 These include the districts of Shusha/Shushi, Khankendi/Stepanakert, Khojavend/Martuni, Khojaly/Ivanyan. It is important to note here that the Nagorno-Karabakh conflict presents a particularly complex case for the use of geographical names as well as for the identification of territorial borders. The names given to the region, its cities, and its adjacent districts have in fact been widely debated by the warring parties (see Brown (1995) and Broers (2019) for comprehensive discussions). To avoid confusion, we here provide the readers with the names given to each district by Azerbaijanis and Armenians, respectively.

4 Today, this region is known in Azeri language as Eastern Zangezur, i.e., Kelbajar, Gubadly, Lachin, Zangilan districts.

5 Both Armenia and Azerbaijan have a mandatory military conscription for all able-bodied men (Armenia Law on Military Service, 2020; Azerbaijan Military Service Act, 2011). In Armenia, draft is required for all men aged 18 + and before reaching age of 27. In Azerbaijan, all male citizens upon their 15^th^ birthday are obliged to register and screen their physical fitness for military service with the State Service for Mobilization and Conscription, to be then called up for military training upon reaching the age of 18. All male citizens automatically become a conscript upon reaching the age of 18.

6 For example, in early October, Armenian authorities claimed to have inflicted as many as 10,000 military casualties to Azerbaijan (Heil, 2020). Similarly, just after a week, the Azerbaijani Ministry of Defense reported on Twitter the killing of over 2,300 Armenian soldiers (Mitra, 2020).

7 Age groups were harmonized due to different countries having slightly different age groups, especially for children and the elderly. For example, Armenia data include ages 0–4, 5–9, and 10–14 while Azerbaijan’s data include ages 0, 1–4, 5–9, and 10–4. Artsakh’s data include ages 0–4, 5–10, and 11–14. The final harmonized age groups are as follows: 0–4, 5–14, 15–19, 20–24, 25–29, 30–34, 35–39, 40–44, 45–49, 50–54, 55–59, 60–64, 65–69, 70–74, 75–79, and 80 + .

8 The over-dispersed Poisson results in point estimates are identical to the regular Poisson, but results in wider confidence intervals which are robust to violation of the mean and variance equality underlying regular Poisson.

9 The one exception to this rule is our prediction for Artsakh, where the OPT model leads to instability in the estimated variance, yielding nonsensical upper bounds for the confidence intervals. For example, females aged 20–24 upper-bound CI is 1,100 deaths (more than double the number of female deaths in Artsakh in 2019, across all ages). Thus, the final prediction and confidence interval for de facto Artsakh does not include the OPT.

10 For example, for males aged 15–19 in Armenia, the OPM prediction is 68 [95% CI 41, 110], the OPT prediction is 43 [12, 160], and the Lee-Carter prediction is 45 [24, 73]. Thus, our final expected number of deaths for this group is 68 with an enveloping interval of [11, 160]. For all model estimates, see Tables A1-A3 in the Supplementary Material.

11 For example, for males aged 15–19 in Armenia, the OPT expectation is 43 [12, 160] using 2015–2019 data and 45 [35, 58] if excluding 2016. Full results are available upon request.

12 Point estimates and confidence intervals for models using death rates, shown in Tables A4-A5 in the Supplementary Material, yielded extremely similar results to the count models. For example, with death counts, our point estimates for 2020 deaths among Armenian males aged 15-19 are 43 (OPT), 45 (Lee-Carter) and 68 (OPM). Using rates, the respective point estimates are 45, 45, and 62.

13 We thank Dmitry Kobak for helping us obtain this data from Russia’s national statistics office.

14 Unfortunately, we cannot estimate the same models for Turkey as it has yet to report 2020 mortality data (Yaman, 2021). A preliminary analysis of age-level mortality data from several cities in Turkey finds no excess in young age groups (Mourenza, 2022).

15 The British International Institute for Strategic Studies (IISS) (2020) estimated the number of personnel in the Armed Forces of Armenia in 2020 to be 41,850 (of which 18,950 conscripts) and in Azerbaijan to be 66,950. If we were to assume that all excess mortality we estimated had occurred among combatants, in 44-days of war, Armenia and Azerbaijan would have lost about 7% and 5% of their military personnel, respectively.

## Declarations

### Data availability

Data for the study are available from the corresponding author or, alternatively, can be downloaded in raw format from the following links: State Statistical Committee of Azerbaijan (2021a). Deaths by sex and age groups 2010–2020. Available at: https://www.stat.gov.az/source/demoqraphy/?lang=en. State Statistical Committee of Azerbaijan (2021b). Demographic indicators of Azerbaijan year-book. Available at: https://www.stat.gov.az/menu/6/statistical_yearbooks/source/demoqraphy_2021.zip. Statistical Committee of Armenia (2021). The Demographic Handbook of Armenia, 2021. Available at: https://www.armstat.am/en/?nid=82&id=2446. National Statistical Service of Nagorno-Karabakh (2021). Statistical Yearbook of Artsakh Republic, 2020. Available at: http://www.stat-nkr.am/en/2010-11-24-11-18-12. Eurostat. (2021). Deaths by week, sex and 5-year age group. Available at: https://ec.europa.eu/eurostat/databrowser/view/demo_r_mwk_05/default/table?lang=en

### Code availability

We are currently working on making the code associated with the current submission available to the public and readers of PRPR on our GitHub repositories and will ensure the process will be completed by the time of publication. Codes and replication files for this study can be found at: https://github.com/akarlinsky/nk-war-2020-excess-mortality.
