# Supplementary material for: The Casualties of War: An Excess Mortality Estimate of Lives Lost in the 2020 Nagorno-Karabakh Conflict
Source: Popul Res Policy Rev. 2023 May 10;42(3):41. doi: 10.1007/s11113-023-09790-2 (PMC10171164; doi:10.1007/s11113-023-09790-2)
Supplement: Supplementary file 1 — Supplementary file1 (DOCX 663 kb) [file 11113_2023_9790_MOESM1_ESM.docx]

# Supplementary Material

## 1. Mortality data

The National Statistical Committees (NSC) of the Republics of Armenia and Azerbaijan are the official demographic and mortality data producers in the two countries. Death counts come from medical certificates, and are annually published by the two institutions as mortality yearbooks. These data are also reported to the WHO (Đoković, 2019; Farrington et al., 2016). PDF and Excel file versions of mortality yearbooks were available from Armenia and Azerbaijan NSCs official websites. In both countries, in-person and online services for registration of births and deaths remained possible during the 2020 Covid-related lockdowns (AbouZahr et al., 2021). In Armenia, data from Eurostat were also available, provided as weekly counts disaggregated by age and sex. Instead, similar data were not available for Azerbaijan and Artsakh, the latter not being recognized as a sovereign entity by the European Union. Most data in Eurostat is by date of occurrence, yet data from the UK and Armenia is by date of registration. For *de facto* Artsakh, we relied on data reported by the National Statistical Committee of the Republic of Nagorno-Karabakh. Since the First Karabakh War, the territory and all its institutions have been considered as independent by Armenia (despite no official recognition). As such, its demographic events and population counts have never been included in Armenia’s statistics (or in Azerbaijan’s), and are collected separately by the Artsakh NSC (Duthé et al., 2010).

Since independence, deaths registered with medical certification have considerably increased in both countries. Nowadays, nearly all deaths in Azerbaijan and all in Armenia are medically certified as part of the civil registration system (UN ESCAP, 2020a-b; WHO, 2016). In terms of coverage, NSCs mortality data quality appears to have improved over time. However, more so in Armenia than in Azerbaijan. While there is evidence that in the early 2000s Armenia’s civil registration system suffered from considerable under-registration of infant and old-age deaths (Duthé et al., 2010; Yeganyan et al., 2001), important steps have been taken since to improve accessibility and coverage, including the establishment of a fully digitalized system for registration of vital demographic events. Recent estimates suggest current death coverage to be over 95% (Wang et al., 2017; UN ESCAP, 2020a). In Azerbaijan, the issue has been historically less studied due to the high level of secrecy surrounding population statistics in the country. Available evidence suggests that completeness levels of death registration have been in the 79–80% range over the period 1990-2014, with underreporting mainly concerning infant (female) deaths (Mikkelsen et al., 2015; Wang et al., 2017; WHO, 2006). While this evidence is reassuring in that our results mainly concern young adult men (and thus overall may represent lower-bound estimates), we are aware that ensuring the accuracy of the data is a prerequisite to any attempt to achieve reliable estimations. Regrettably, at the time of writing, no data sources were available to the researchers to further evaluate the coverage and quality of Azerbaijan’s vital system data. We have requested access to samples of census data, which could have been used to examine adult mortality coverage using the General Growth Balance method or/and the Synthetic Extinct Generations method. However, these were not made available in any format to the researchers. Alternative data sources are also scant, with only one Demographic and Health Survey (implemented in 2006) available for the country.

## 2. Causes of death

Unfortunately, neither Armenia’s nor Azerbaijan’s Demographic Yearbooks provided sufficient age-sex disaggregated data to examine causes of death for 2020, as well as for the preceding five-year window. Moreover, while Armenia categorized separately deaths due to war violence (in aggregate format without age-sex disaggregation), Azerbaijan did not differentiate them from other ‘external causes’ (including road accidents, poisoning and injuries), thereby making it hard to systematically analyze and compare changes in causes of death across and within populations. Nevertheless, to examine changes in other causes of death that may provide alternative explanations for the excess losses we estimate, we use 2015-2020 data on annual traffic accident deaths by sex from the United Nations Economic Commission for Europe (UNECE) (2022) and on homicide victims by sex from the United Nations Office on Drugs and Crime (UNODC) (2022). Figure A1 presents the results. In both Armenia and Azerbaijan, male and female deaths from homicides and traffic accidents are almost completely in line with previous years, in some cases (e.g., total deaths from road traffic accidents among Azerbaijani men) even decreasing slightly. Thus, even if all deaths from these causes were in our conflict-affected age-groups, changes in these causes are miniscule in comparison to the high excess mortality caused by the conflict.


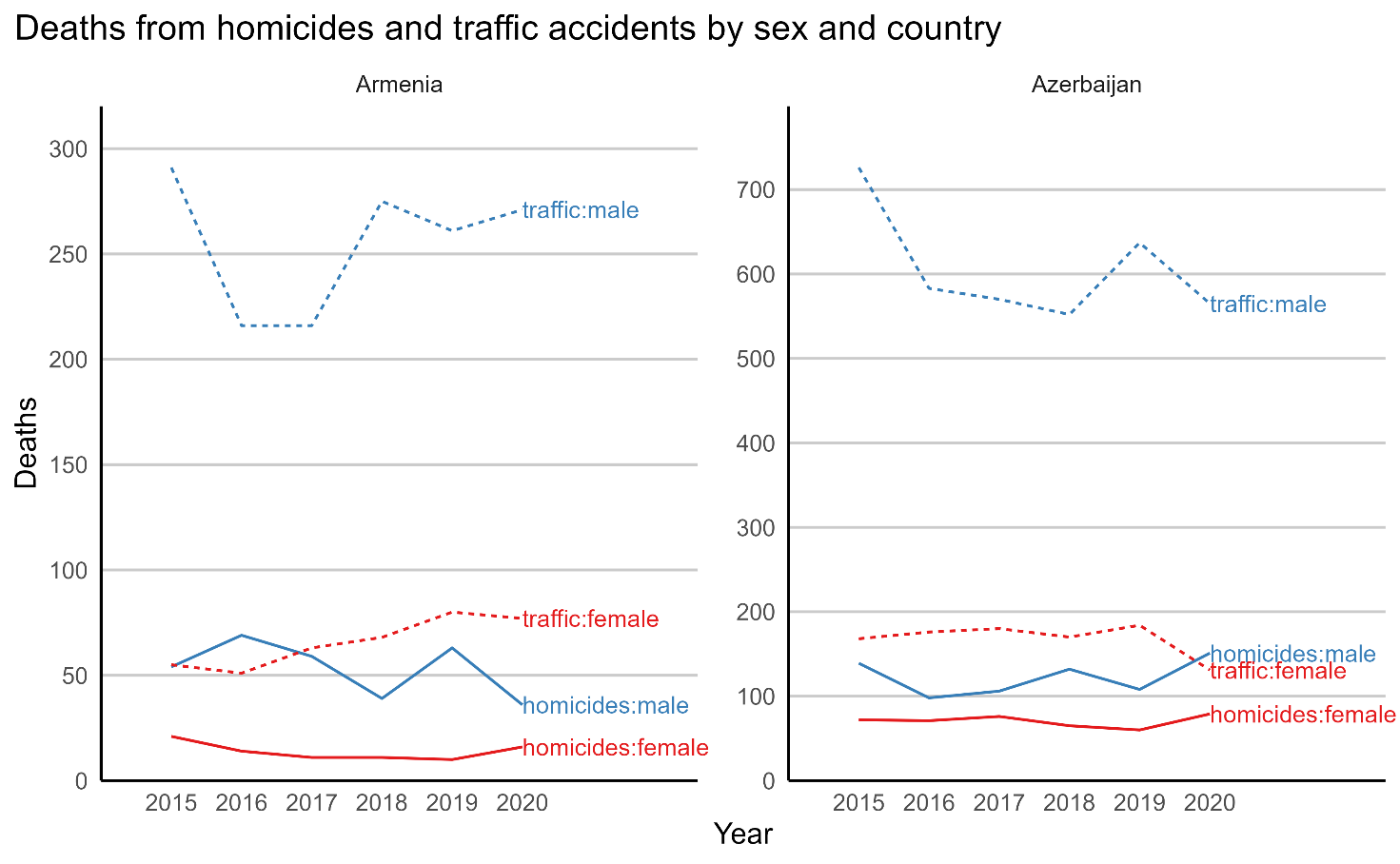
**Figure A1. Homicide and road traffic deaths by sex in Armenia and Azerbaijan, 2015-2020**

Source: UNECE (2022) for road traffic deaths UNODC (2022) for homicides.

## 3. Comparison of Eurostat’s data on weekly deaths by age and sex


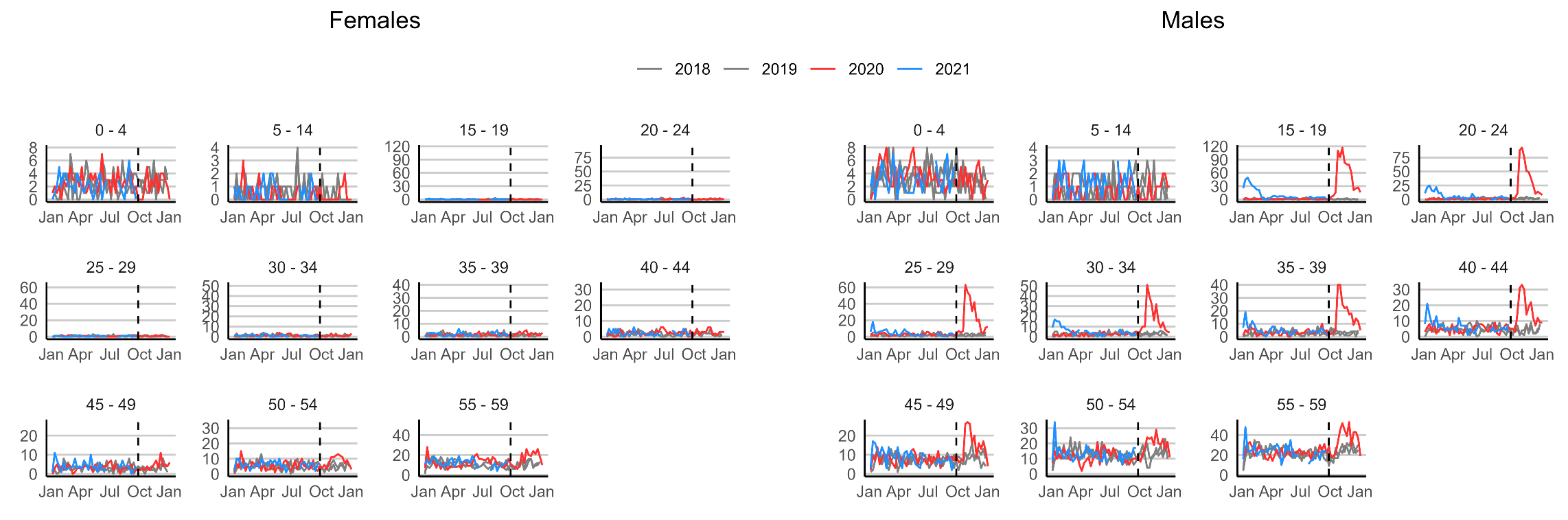
**Figure A2. Weekly deaths by age-group and sex, Armenia (including deaths registered until March 2021)**

Source: Eurostat (2021). Note: the dashed line indicates 27 September 2020 (war onset). The y-axes differ across age-groups, but are the same for men and women belonging to the same age category.


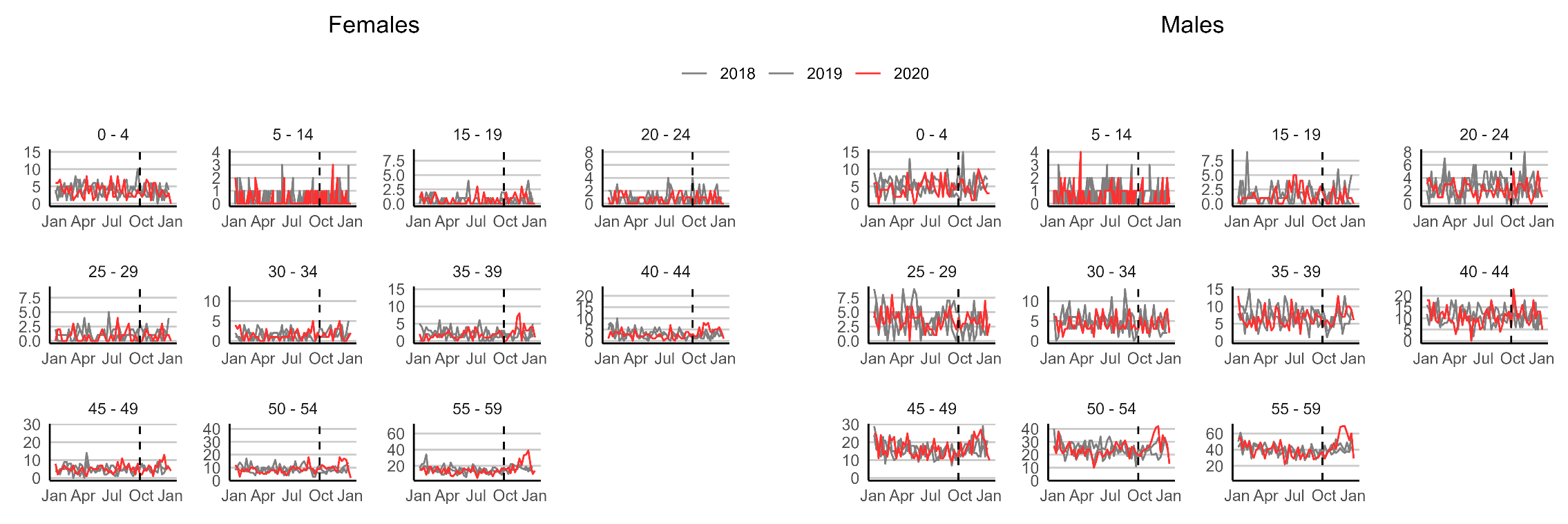
**Figure A3. Weekly deaths by age-group and sex, Georgia**

Source: GeoStat (2021). Note: the dashed line indicates 27 September 2020 (onset of the Second Karabakh War). The y-axes differ across age-groups, but are the same for men and women belonging to the same age category.

## 4. Full predictions from estimated models

**Table A1 Expected mortality by age group and sex from estimated models, Armenia**

| **Age Group** | | **Male** | | | | **Female** | | |
| --- | --- | --- | --- | --- | --- | --- | --- | --- |
|  |  | **OPT** | **OPM** | **Lee-Carter** | | **OPT** | **OPM** | **Lee-Carter** |
| 0 – 4 |  | 139 | 206 | 159 |  | 110 | 152 | 124 |
|  |  | [113, 171] | [160, 266] | [105, 219] |  | [97, 124] | [124, 187] | [87, 163] |
| 5 – 14 |  | 39 | 46 | 41 |  | 30 | 32 | 31 |
|  |  | [23, 65] | [38, 56] | [27, 56] |  | [22, 40] | [28, 35] | [21, 42] |
| 15 – 19 |  | 43 | 68 | 45 |  | 18 | 16 | 18 |
|  |  | [12, 160] | [41, 110] | [24, 73] |  | [10, 34] | [12, 20] | [11, 28] |
| 20 – 24 |  | 59 | 95 | 70 |  | 24 | 30 | 26 |
|  |  | [39, 89] | [69, 131] | [43, 104] |  | [15, 36] | [24, 36] | [17, 38] |
| 25 – 29 |  | 89 | 113 | 98 |  | 36 | 38 | 36 |
|  |  | [60, 134] | [93, 136] | [75, 124] |  | [22, 57] | [32, 44] | [23, 50] |
| 30 – 34 |  | 127 | 137 | 131 |  | 52 | 54 | 53 |
|  |  | [96, 167] | [124, 152] | [112, 159] |  | [41, 65] | [50, 59] | [37, 74] |
| 35 – 39 |  | 155 | 162 | 158 |  | 61 | 74 | 61 |
|  |  | [129, 186] | [152, 173] | [134, 178] |  | [32, 117] | [59, 94] | [41, 85] |
| 40 – 44 |  | 225 | 255 | 236 |  | 71 | 101 | 77 |
|  |  | [179, 282] | [229, 285] | [201, 276] |  | [47, 105] | [78, 130] | [45, 108] |
| 45 – 49 |  | 368 | 396 | 381 |  | 130 | 174 | 139 |
|  |  | [254, 533] | [349, 450] | [343, 421] |  | [85, 200] | [138, 219] | [95, 194] |
| 50 – 54 |  | 595 | 768 | 654 |  | 228 | 314 | 257 |
|  |  | [548, 646] | [652, 905] | [529, 807] |  | [201, 258] | [255, 386] | [186, 348] |
| 55 – 59 |  | 1165 | 1280 | 1201 |  | 543 | 592 | 555 |
|  |  | [1066, 1273] | [1197, 1369] | [1073, 1318] |  | [479, 615] | [553, 635] | [499, 631] |
| 60 – 64 |  | 1577 | 1529 | 1533 |  | 837 | 807 | 830 |
|  |  | [1344, 1851] | [1446, 1617] | [1459, 1607] |  | [754, 929] | [774, 841] | [768, 889] |
| 65 – 69 |  | 1704 | 1536 | 1616 |  | 1074 | 1001 | 1043 |
|  |  | [1465, 1984] | [1411, 1672] | [1490, 1767] |  | [1000, 1153] | [950, 1054] | [945, 1152] |
| 70 – 74 |  | 1326 | 1168 | 1276 |  | 1023 | 944 | 978 |
|  |  | [955, 1840] | [1018, 1340] | [1121, 1474] |  | [719, 1454] | [830, 1073] | [901, 1068] |
| 75 – 79 |  | 1317 | 2068 | 1544 |  | 1555 | 2263 | 1780 |
|  |  | [1038, 1670] | [1557, 2747] | [1039, 2102] |  | [1229, 1969] | [1778, 2879] | [1291, 2430] |
| 80+ |  | 4080 | 3990 | 4003 |  | 6819 | 6633 | 6793 |
|  |  | [3551, 4688] | [3806, 4183] | [3877, 4120] |  | [6205, 7493] | [6401, 6873] | [6548, 7066] |

Source: Armenia National Demographic Yearbook (Statistical Committee of Armenia, 2021)

**Table A2 Expected mortality by age group and sex from estimated models, Azerbaijan**

| **Age Group** | | **Male** | | | | **Female** | | |
| --- | --- | --- | --- | --- | --- | --- | --- | --- |
|  |  | **OPT** | **OPM** | **Lee-Carter** | | **OPT** | **OPM** | **Lee-Carter** |
| 0 – 4 |  | 989 | 1212 | 1059 |  | 667 | 829 | 712 |
|  |  | [837, 1169] | [1053, 1394] | [912, 1206] |  | [570, 780] | [715, 962] | [607, 820] |
| 5 – 14 |  | 311 | 255 | 290 |  | 191 | 164 | 183 |
|  |  | [290, 334] | [222, 292] | [234, 340] |  | [152, 241] | [144, 187] | [150, 210] |
| 15 – 19 |  | 218 | 220 | 218 |  | 103 | 108 | 105 |
|  |  | [159, 298] | [199, 243] | [187, 251] |  | [68, 154] | [95, 123] | [86, 128] |
| 20 – 24 |  | 269 | 337 | 291 |  | 129 | 160 | 135 |
|  |  | [188, 386] | [281, 405] | [251, 351] |  | [96, 174] | [136, 188] | [107, 173] |
| 25 – 29 |  | 394 | 405 | 397 |  | 188 | 199 | 193 |
|  |  | [344, 452] | [386, 424] | [360, 434] |  | [151, 234] | [184, 216] | [165, 224] |
| 30 – 34 |  | 495 | 478 | 489 |  | 248 | 229 | 242 |
|  |  | [376, 652] | [436, 524] | [442, 526] |  | [206, 299] | [211, 248] | [207, 284] |
| 35 – 39 |  | 574 | 589 | 577 |  | 303 | 271 | 296 |
|  |  | [420, 786] | [532, 652] | [536, 623] |  | [192, 479] | [229, 320] | [250, 344] |
| 40 – 44 |  | 873 | 836 | 860 |  | 374 | 367 | 374 |
|  |  | [815, 936] | [805, 867] | [788, 920] |  | [290, 481] | [338, 398] | [328, 416] |
| 45 – 49 |  | 1295 | 1375 | 1321 |  | 565 | 618 | 580 |
|  |  | [1071, 1567] | [1279, 1479] | [1234, 1411] |  | [484, 659] | [572, 667] | [528, 632] |
| 50 – 54 |  | 1984 | 2266 | 2069 |  | 1017 | 1120 | 1041 |
|  |  | [1740, 2261] | [2062, 2490] | [1885, 2223] |  | [941, 1099] | [1048, 1197] | [944, 1126] |
| 55 – 59 |  | 3483 | 3335 | 3431 |  | 1808 | 1755 | 1792 |
|  |  | [3121, 3887] | [3186, 3491] | [3299, 3606] |  | [1707, 1916] | [1708, 1803] | [1695, 1882] |
| 60 – 64 |  | 4259 | 3550 | 4003 |  | 2444 | 2125 | 2346 |
|  |  | [3976, 4563] | [3139, 4014] | [3558, 4601] |  | [2268, 2634] | [1931, 2338] | [2125, 2640] |
| 65 – 69 |  | 3883 | 3179 | 3635 |  | 2848 | 2302 | 2679 |
|  |  | [3196, 4718] | [2748, 3679] | [3142, 4175] |  | [2379, 3410] | [1977, 2681] | [2323, 3119] |
| 70 – 74 |  | 2399 | 2174 | 2315 |  | 2158 | 1972 | 2114 |
|  |  | [1890, 3046] | [1963, 2408] | [2149, 2494] |  | [1649, 2824] | [1771, 2195] | [1961, 2307] |
| 75 – 79 |  | 2633 | 3678 | 2889 |  | 2870 | 4265 | 3102 |
|  |  | [1949, 3558] | [2942, 4599] | [2239, 3552] |  | [2178, 3782] | [3303, 5508] | [2289, 3984] |
| 80+ |  | 6983 | 6122 | 6683 |  | 10978 | 9830 | 10589 |
|  |  | [5825, 8372] | [5510, 6802] | [6122, 7312] |  | [9544, 12629] | [9023, 10710] | [9856, 11376] |

Source: Azerbaijan Demographic Yearbook (Statistical Committee of Azerbaijan, 2021a-b).

**Table A3 Expected mortality by age group and sex from estimated models, *de facto* Artsakh**

| **Age Group** | | **Male** | | | | **Female** | | |
| --- | --- | --- | --- | --- | --- | --- | --- | --- |
|  |  | **OPT** | **OPM** | **Lee-Carter** | | **OPT** | **OPM** | **Lee-Carter** |
| 0 – 4 |  | - | 13 | 11 |  | - | 14 | 16 |
|  |  |  | [9, 19] | [3, 22] |  |  | [9, 21] | [6, 33] |
| 5 – 14 |  | - | 3 | 3 |  | - | 1 | 2 |
|  |  |  | [2, 7] | [0, 8] |  |  | [1, 3] | [0, 9] |
| 15 – 19 |  | - | 5 | 5 |  | - | 2 | 2 |
|  |  |  | [3, 8] | [1, 11] |  |  | [1, 4] | [0, 5] |
| 20 – 24 |  | - | 6 | 4 |  | - | 0 | 0 |
|  |  |  | [3, 11] | [0, 14] |  |  | [0, 3] | [0, 3] |
| 25 – 29 |  | - | 6 | 6 |  | - | 2 | 3 |
|  |  |  | [5, 8] | [1, 12] |  |  | [1, 6] | [0, 10] |
| 30 – 34 |  | - | 7 | 5 |  | - | 2 | 3 |
|  |  |  | [4, 11] | [1, 14] |  |  | [1, 5] | [0, 24] |
| 35 – 39 |  | - | 12 | 9 |  | - | 3 | 2 |
|  |  |  | [6, 21] | [1, 23] |  |  | [1, 7] | [0, 13] |
| 40 – 44 |  | - | 13 | 10 |  | - | 5 | 4 |
|  |  |  | [8, 21] | [3, 23] |  |  | [3, 9] | [0, 8] |
| 45 – 49 |  | - | 17 | 18 |  | - | 7 | 8 |
|  |  |  | [12, 22] | [10, 32] |  |  | [5, 10] | [2, 19] |
| 50 – 54 |  | - | 27 | 26 |  | - | 12 | 11 |
|  |  |  | [23, 31] | [16, 35] |  |  | [9, 17] | [7, 17] |
| 55 – 59 |  | - | 44 | 46 |  | - | 23 | 23 |
|  |  |  | [38, 50] | [31, 68] |  |  | [17, 31] | [14, 36] |
| 60 – 64 |  | - | 54 | 51 |  | - | 28 | 31 |
|  |  |  | [45, 65] | [37, 70] |  |  | [23, 35] | [15, 55] |
| 65 – 69 |  | - | 56 | 55 |  | - | 32 | 31 |
|  |  |  | [50, 61] | [40, 73] |  |  | [28, 38] | [20, 39] |
| 70 – 74 |  | - | 49 | 57 |  | - | 30 | 30 |
|  |  |  | [34, 70] | [23, 147] |  |  | [25, 34] | [21, 42] |
| 75 – 79 |  | - | 75 | 66 |  | - | 71 | 77 |
|  |  |  | [55, 104] | [40, 100] |  |  | [48, 105] | [42, 137] |
| 80+ |  | - | 241 | 248 |  | - | 359 | 355 |
|  |  |  | [222, 261] | [202, 289] |  |  | [334, 386] | [311, 389] |

Source: National Statistical Service of Nagorno-Karabakh (2021).

**Table A4 Expected mortality by age group and sex, models estimated on death rates, Armenia**

| **Age Group** | | **Male** | | | | **Female** | | |
| --- | --- | --- | --- | --- | --- | --- | --- | --- |
|  |  | **Poisson Trend** | **Poisson Mean** | **Lee-Carter** | | **Poisson Trend** | **Poisson Mean** | **Lee-Carter** |
| 0 – 4 |  | 132 | 189 | 154 |  | 104 | 141 | 116 |
|  |  | [110,157] | [150,238] | [105,204] |  | [93,117] | [116,171] | [84,147] |
| 5 – 14 |  | 39 | 48 | 42 |  | 30 | 33 | 32 |
|  |  | [23,65] | [39,60] | [33,51] |  | [22,40] | [30,38] | [29,34] |
| 15 – 19 |  | 45 | 62 | 45 |  | 19 | 14 | 18 |
|  |  | [12,169] | [40,97] | [22,68] |  | [10,37] | [11,19] | [13,23] |
| 20 – 24 |  | 61 | 75 | 65 |  | 24 | 23 | 24 |
|  |  | [41,91] | [63,90] | [50,79] |  | [16,36] | [20,26] | [22,26] |
| 25 – 29 |  | 83 | 104 | 94 |  | 33 | 34 | 33 |
|  |  | [57,122] | [87,125] | [78,108] |  | [22,51] | [30,39] | [32,34] |
| 30 – 34 |  | 123 | 147 | 135 |  | 50 | 57 | 54 |
|  |  | [94,161] | [127,171] | [115,153] |  | [40,63] | [51,64] | [48,58] |
| 35 – 39 |  | 160 | 183 | 172 |  | 63 | 84 | 65 |
|  |  | [131,194] | [164,204] | [154,189] |  | [33,120] | [64,110] | [40,89] |
| 40 – 44 |  | 236 | 258 | 244 |  | 73 | 103 | 79 |
|  |  | [191,293] | [236,282] | [222,264] |  | [48,109] | [80,132] | [49,108] |
| 45 – 49 |  | 355 | 353 | 353 |  | 134 | 165 | 139 |
|  |  | [243,520] | [313,399] | [352,355] |  | [90,200] | [138,198] | [107,170] |
| 50 – 54 |  | 629 | 628 | 624 |  | 225 | 258 | 236 |
|  |  | [585,678] | [614,642] | [617,630] |  | [194,261] | [234,283] | [209,261] |
| 55 – 59 |  | 1062 | 1181 | 1111 |  | 507 | 584 | 531 |
|  |  | [941,1198] | [1092,1277] | [1002,1213] |  | [457,563] | [530,644] | [459,599] |
| 60 – 64 |  | 1588 | 1789 | 1661 |  | 840 | 940 | 886 |
|  |  | [1364,1849] | [1629,1965] | [1455,1858] |  | [760,928] | [866,1021] | [808,958] |
| 65 – 69 |  | 1652 | 1855 | 1741 |  | 1032 | 1231 | 1102 |
|  |  | [1446,1887] | [1697,2029] | [1547,1923] |  | [951,1121] | [1089,1391] | [924,1278] |
| 70 – 74 |  | 1602 | 1570 | 1594 |  | 1277 | 1283 | 1292 |
|  |  | [1486,1727] | [1525,1616] | [1559,1635] |  | [1104,1477] | [1221,1347] | [1282,1304] |
| 75 – 79 |  | 1086 | 1266 | 1165 |  | 1269 | 1443 | 1333 |
|  |  | [1011,1167] | [1155,1388] | [1029,1295] |  | [1216,1325] | [1336,1560] | [1197,1460] |
| 80+ |  | 4033 | 4221 | 4079 |  | 6743 | 7606 | 7143 |
|  |  | [3455,4708] | [3981,4475] | [3844,4289] |  | [6036,7533] | [6965,8305] | [6487,7754] |

Source: Armenia National Demographic Yearbook (Statistical Committee of Armenia, 2021)

**Table A5 Expected mortality by age group and sex, models estimated on death rates, Azerbaijan**

| **Age Group** | | **Male** | | | | **Female** | | |
| --- | --- | --- | --- | --- | --- | --- | --- | --- |
|  |  | **Poisson Trend** | **Poisson Mean** | **Lee-Carter** | | **Poisson Trend** | **Poisson Mean** | **Lee-Carter** |
| 0 – 4 |  | 945 | 1055 | 988 |  | 637 | 729 | 668 |
|  |  | [751,1191] | [955,1166] | [890,1077] |  | [525,775] | [656,809] | [615,716] |
| 5 – 14 |  | 308 | 281 | 298 |  | 190 | 180 | 188 |
|  |  | [287,331] | [263,301] | [274,327] |  | [151,240] | [165,196] | [181,197] |
| 15 – 19 |  | 229 | 213 | 227 |  | 108 | 102 | 110 |
|  |  | [166,317] | [191,239] | [208,251] |  | [75,155] | [90,116] | [103,117] |
| 20 – 24 |  | 265 | 297 | 289 |  | 128 | 137 | 127 |
|  |  | [182,385] | [259,340] | [278,298] |  | [96,169] | [124,151] | [118,134] |
| 25 – 29 |  | 386 | 394 | 385 |  | 183 | 188 | 188 |
|  |  | [334,446] | [376,413] | [371,397] |  | [144,233] | [174,204] | [187,188] |
| 30 – 34 |  | 488 | 512 | 486 |  | 243 | 245 | 241 |
|  |  | [370,645] | [465,563] | [446,521] |  | [203,292] | [231,260] | [237,245] |
| 35 – 39 |  | 569 | 652 | 583 |  | 303 | 302 | 301 |
|  |  | [414,782] | [569,748] | [481,680] |  | [192,479] | [260,351] | [301,302] |
| 40 – 44 |  | 905 | 903 | 898 |  | 387 | 385 | 392 |
|  |  | [814,1006] | [872,934] | [890,905] |  | [310,482] | [358,413] | [386,399] |
| 45 – 49 |  | 1290 | 1327 | 1276 |  | 561 | 590 | 575 |
|  |  | [1066,1562] | [1245,1414] | [1199,1344] |  | [477,660] | [555,626] | [561,587] |
| 50 – 54 |  | 1987 | 2227 | 2049 |  | 1009 | 1109 | 1025 |
|  |  | [1742,2268] | [2049,2420] | [1794,2290] |  | [943,1081] | [1040,1182] | [950,1093] |
| 55 – 59 |  | 3295 | 3612 | 3391 |  | 1723 | 1927 | 1775 |
|  |  | [3077,3529] | [3386,3854] | [3052,3707] |  | [1628,1822] | [1785,2081] | [1633,1904] |
| 60 – 64 |  | 4281 | 4596 | 4397 |  | 2448 | 2726 | 2500 |
|  |  | [3920,4676] | [4343,4864] | [4071,4695] |  | [2243,2672] | [2520,2949] | [2285,2697] |
| 65 – 69 |  | 3740 | 4104 | 3896 |  | 2737 | 2937 | 2763 |
|  |  | [3257,4293] | [3794,4439] | [3557,4209] |  | [2423,3092] | [2758,3128] | [2594,2915] |
| 70 – 74 |  | 2908 | 2861 | 2894 |  | 2601 | 2555 | 2589 |
|  |  | [2697,3135] | [2783,2941] | [2849,2949] |  | [2492,2715] | [2507,2603] | [2560,2623] |
| 75 – 79 |  | 2408 | 2643 | 2453 |  | 2576 | 3137 | 2642 |
|  |  | [2020,2870] | [2450,2852] | [2202,2689] |  | [2251,2947] | [2772,3550] | [2264,3013] |
| 80+ |  | 6842 | 6797 | 6942 |  | 10644 | 11358 | 10716 |
|  |  | [5735,8163] | [6412,7206] | [6734,7195] |  | [9335,12136] | [10683,12075] | [10109,11261] |

Source: Azerbaijan Demographic Yearbook (Statistical Committee of Azerbaijan, 2021a-b).
